# Beyond Beautiful: Embroidering Legible and Expressive Tactile Graphics

**DOI:** 10.1145/3663547.3746336

**Published:** 2025

**Authors:** Margaret Ellen Seehorn, Claris Winston, Bo Liu, Gene S-H Kim, Emily White, Nupur Gorkar, Kate S Glazko, Aashaka Desai, Jerry Cao, Megan Hofmann, Jennifer Mankoff

**Affiliations:** University of Michigan, Ann Arbor, Michigan, USA; University of Washington, Seattle, Washington, USA; Cornell University, New York, New York, USA; Stanford University, Stanford University, Stanford, California, USA; University of Washington, Seattle, Washington, USA; University of Washington, Seattle, Washington, USA; University of Washington, Seattle, Washington, USA; Paul G. Allen School of Computer Science and Engineering, University of Washington, Seattle, Washington, USA; Paul G. Allen School of Computer Science & Engineering, University of Washington, Seattle, Washington, USA; Khoury College of Computer Sciences, Northeastern University, Boston, Massachusetts, USA; Allen School of Computer Science and Engineering, University of Washington, Seattle, Washington, USA

**Keywords:** Machine Embroidery, Tactile Graphics, Blind and Visually Impaired

## Abstract

Tactile graphics present visual information to blind and visually-impaired individuals in an accessible way, through touch. Current methods for producing tactile graphics, such as embossing or swell-paper printing, have limitations such as durability – and the tools required to produce them are limited in expressiveness. In this project, we explore embroidery as a medium for producing tactile graphics. Embroidery, traditionally known for its variety and visual beauty, offers not just improved durability and ease of production – but the ability to convey information through a broad range of stitch types. Following an exploration of the design space of embroidered tactile graphics, we identify key perceptual properties that impact how embroidered textures are differentiated. Based on these differences, we introduce an optimization algorithm for assigning textures to regions of tactile graphics in a way that makes them diverse and legible. We implement an end-to-end pipeline for producing embroidered tactile graphics and evaluate the comprehensibility and legibility of our design with 6 blind participants. Our findings showed that embroidered tactile graphics present information accurately and comprehensively, and that measurable properties, such as the use of spacing and distinctiveness, were an important factor of expressive and legible design.

## 1 Introduction

Tactile graphics are tangible representations of visual information that consist of raised lines, textures, Braille, and other tactile features that can be interpreted non-visually by blind or visually impaired (BVI) users [22]. Tactile graphics are first prepared by simplifying a source image, to which textures are applied and then rendered, typically using specialized technology such as Braille embossers or swell paper [68]. However, tools for creating tactile graphics may be limited in their availability, with a small range of printing options suitable for personal use– many of which remain prohibitively expensive in low-resource settings [21, 27]. As a result, sometimes tactile graphics are found only in institutional settings such as universities or available by-order through the mail. Some work has begun to improve the availability of embossed materials [18]. However, even if widely available, embossed card stock or swell-paper may degrade over time or when folded [68, 94]. While state-of-the-art technologies such as electronic Braille readers are becoming capable of rendering tactile graphics [4], they are cost-prohibitive and limited in their ability to convey diverse textures outside of the dot-based grid system.

Tactile graphics made with cloth represent a complementary, durable alternative. Already, embroidery and fabric are popular materials for children’s books regardless of sensory disabilities because they are durable–withstanding active play, and safe to interact with due to their soft nature (e.g., [46]). Embroidery, traditionally valued for its beauty and cultural relevance [81], represents an ubiquitous, under explored production medium for creating tactile graphics. While embroidery is much slower to create–whether hand or machine-embroidered–it offers material durability [84], beauty, and tactile expressiveness: allowing for various stitch textures, materials, and colors, resulting in haptically rich and multi-modal graphics. Indeed, emerging works in domains outside of accessibility have already begun to leverage embroidery as a medium for data visualization physicalization for such properties [65, 84, 91]. And within the domain of accessibility, researchers have begun to explore the potential for embroidery to convey context-rich information across domains such as visualizing graphs or historical artifacts [53, 78]. Importantly, embroidery leverages a widespread and culturally-embedded skill set [14, 81]– found among educators [74], artists, and hobbyists– which enables a broader set of contributors to participate in the creation of accessible materials.

The application of embroidery to the creation of tactile graphics, while emergent in domain-specific areas such as data visualization [78] and historic artifacts [53], remains largely unexplored, with few established best practices or design guidelines for ensuring tactile richness and legibility. To do so, we build on the only prior work in embroidered tactile graphic creation [78], which introduces a method for creating embroidered Braille, an optimization for assigning textures to regions of a tactile diagram, and presents qualitative data showing that BVI users like the resulting diagrams about as much as embossed graphics. While Seehorn et al. demonstrate that embroidered tactile graphics are potentially valuable [78], the current work provides guidance on how to design tactile graphics that utilize embroidery’s material affordances (e.g. stitch variation, texture). As with that prior work, we are not automating the *simplification* of graphics for tactile use; our work focuses on automated methods for assigning textures to them using embroidery. We also expand the prior work with support for essential features such as lines; describe an end-to-end manufacturing pipeline; support integrated printing of Braille^1^; and provide quantitative data on legibility of tactile graphics. Our contributions are as follows:

**Manual Design of Tactile Graphics for Machine Embroidery** Regardless of the means of production, a principal challenge is to design tactile graphics using the affordances of these techniques. Emerging work describes some design considerations for embroidery as a medium for communicating graphics such as data visualizations [2, 84], but these are typically described for visual appeal rather than tactile clarity. We present manually developed machine-embroidered tactile graphics in two domains: A young children’s book, and scientific diagrams. Based on this we discuss design requirements for embroidered tactile graphics.

**Categorization of Texture and Automatic Assignment** To support tactile design, a system must enable the creation of embroidery patterns that prioritize tactile legibility and haptic distinctiveness, rather than relying solely on aesthetics intended for sighted audiences. We expand on [78], which only supports filled (textured) regions, by adding a new category of visual elements lines. We collected metrics about textured lines and regions and developed an optimization algorithm for assigning textures to figures that include lines, regions, and borders (around regions).

**Printing Pipeline** While the production of embroidered graphics and books can be completed with a variety of tools (e.g., automatic embroidery machines, manual embroidery machines, by expert hands), machine embroidery is the fastest. However, printing and assembly is not straightforward given the need to reverse the fabric (prior work simply glued separate Braille labels in place [78]). To improve the machine embroidery experience, we developed an integrated pipeline for reliably producing embroidered tactile graphics with Braille, which takes about 4 hours to produce a single graphic from start to finish, using an embroidery machine costing about $3000.

After providing background information on both tactile graphics, and machine embroidery, Section 3 describes our manual design exploration of machine embroidered tactile graphics with machine-embroidered Braille text. Following that work, Section 4 and Section 5 describe our automated method for assigning embroidery textures to tactile diagrams. Section 6 describes the pipeline for printing graphics with Braille labels. As described in Section 7, we evaluated the resulting graphics in a study exploring the legibility of tactile embroidered diagrams. The results from our user study with BVI individuals shows that embroidered tactile graphics are legible and comprehensible by participants. Feedback from participants also points to important design considerations around deliberate and distinctive textures, and areas for future work to further improve embroidered tactile graphics, especially for more complex graphics.

## 2 Background

This work bridges two largely distinct domains: the design and production of tactile graphics for blind and visually-impaired (BVI) audiences, and the practice of machine embroidery for producing artifacts. First, we describe existing processes for the production of tactile graphics, and some existing spaces where research and innovation is taking place in that domain. Next, we describe challenges to interpretability that arise from these methods, grounded in the theory of tactile semiotics. Then, we describe pathways to improve tactile understanding, grounded in tactile semiotics and existing standards of tactile graphic construction. Finally, we describe relevant terminology and processes used to create machine embroidery, focusing on physical properties relevant for creating tactile graphics and emerging use cases in physical computing and accessibility. Across all of these domains, we highlight areas where more research is needed to inform creation of embroidered tactile graphics.

### 2.1 Tactile Graphics

#### 2.1.1 Producing tactile graphics.

Tactile graphic design has well established guidelines [59], though tactile graphic design is a topic of ongoing study [68, 76]. The pipeline of tactile graphic design typically starts with converting images into a format appropriate for tactile representation, and then producing those graphics using one of a variety of methods/media. In the first step an image or diagram must be *simplified* into a format that is appropriate for tactile understanding. Automation of this step is still an open problem [68], though early work has explored general solutions (e.g., [44]).

Once a graphic has been designed, it needs to be printed or rendered in tactile form [68]. Most tactile graphics are embossed or made with swell paper. Braille embossers [27] require that the source file of an image be converted to an embossing file format, which specifies dot height, size, and spacing to be punctured on heavy-weight paper. Swell paper graphics are made on capsule paper, which is heated to create raised lines and surfaces. Both of these approaches are prohibitively expensive in low-resource settings [20]. Prior work in low-resource settings has explored alternatives to existing methods for tactile graphic production. For example, a Braille character printer can be used to create images [20] or create stencils using a low-cost cutting machine and use them to emboss paper [97].

Some additional work has explored a variety of new media for output [62], such as 3D printing and 2D active displays. For example, Kim and Yeh have created 3D-printed movable tactile picture books for blind children [50]. Most recently, embroidery has been added to this body of work [53, 78]. However, there are still many questions about how to design with embroidery; how to reliably manufacture embroidered tactile graphics; and how legible embroidered tactile graphics are.

#### 2.1.2 Challenges to tactile graphic comprehension.

In the process of simplifying the format of an image or diagram, most existing tactile graphic production methods limit the diversity of textures that can be rendered [60] and minimizes the set of tactile graphics that can be produced [13]. As a result, unmodified tactile graphics often fail to meet user expectations of comprehensibility [76]. Facilitating clear, effective tactile communication is not only a matter of understanding tactile contrast and placement, but also of recognizing and utilizing the meanings ascribed to different types of tactile sensations, as described by the theory of tactile semiotics [35]. Tactile semiotics draws on the principle underlying earlier semiotics—such as linguistic [8] and aesthetic [47]—that every media channel has a set of rules or encodings for communicating meaning that can inform design parameters [35]. The restrictions on tactile range imposed by mainstream tactile graphic production techniques are liable to truncate, omit, or obfuscate relevant information [79]. This violates these semiotic parameters and contributes to the failure to meet expectations of graphic comprehensibility.

#### 2.1.3 Techniques for improving tactile comprehension.

A number of studies conducted to understand the nature of sensory encodings reveal criterion for effective tactile understanding [35, 36, 49]. Formative work in tactile design and sensemaking finds that tactile associations need to be shared to facilitate tactic understanding, and that the most universal associations tend to be formed by the physical nature of the tactile object, both in the sense that an object’s concrete physical properties (e.g. tree fungus perceived as “rugged” versus “delicate”), which allows for common understanding through touch. Such shared understandings of real-world properties of natural/organic materials converged across individuals more readily than representative synthetic materials [35].

Studies evaluating tactile understanding with particular attention to the BVI community add nuance and additional, potentially-contradictory criteria to tactile sense-making. For example, even as precisely representing an object’s physical properties produces common tactile understanding, Gupta et al. finds that simplified two-dimensional shapes facilitate information and recall more effectively than detailed, “visually correct” representations [36]. Though forced tactile oversimplification can contribute to poor comprehensibility [76, 79], simplification is not antithetical to designing for tactile comprehension. Rather, overloading tactile graphics with too much information also is a challenge to comprehensibility [13]. Thus, part of the art of creating tactile graphics is the simplification of the image source file, leading to a line of research that has explored automating the conversion of visual information to a format suitable for tactile graphics (e.g., simplifying, posterizing, projecting 3D models into 2D space) [44, 62, 66].

A few works have looked at automating parts of this process (e.g., [20, 44, 66]). Machine learning techniques have been used to assist in creating tactile graphics from a source image by classifying the graphic, parsing the image into distinct regions and text blocks, and simplifying the image [62]. Optimization has been used to enhance tactile graphics, for example tuning the representation of information in tactile maps [42], and converting 3D models to 2D representations [66]. However much remains to be done before this stage of tactile graphics can reliably be automated across the full range of potential images.

Looking beyond simplification, semiotically motivated works such as Kennedy et al. emphasize the importance of including specific, representative attributes such as borders and outlines, to help with navigation and understanding of tactile illustrations[49]. Similar to simplification, work has been done towards automating the inclusion of borders and outlines in multiple graphic modalities, including raised-line illustrations[54], refreshable Braille displays [67], and, recently, embroidered patches [52]. Principles of tactile semiotics have also been applied in a variety of information communication contexts, especially those with an emphasis on expressivity, such as multi-sensory representations of visual art [7], design of science education materials for young children [5], and tactile drawing [48].

### 2.2 Machine and Automatic Embroidery

Machine embroidery has rarely been used to create tactile graphics, however, the utility of embroidery as a medium for data physicalization is increasingly described [64, 84, 91]. Machine embroidery typically involves attaching a backing to fabric that is then stretched in a hoop frame that holds the fabric during printing. The machine then lays down stitches, creating patterns or textures. Embroidery also has the potential to be used to do more than lay down thread using different stitch patterns by incorporating other materials and techniques (e.g., [26, 37]). Below we describe a typical machine embroidery pipeline and some of the research explorations that have been done with respect to that pipeline.

The typical machine embroidery pipeline starts with selecting a fabric. More detailed treatises that talk about specific material options have been published elsewhere (e.g., [23, 69]). The fabric is often attached to a stiff backing using glue or other methods. Backings are selected for multiple properties, such as specific stiffness or water-solubility. As with hand embroidery, most machine embroidery is done interior to an embroidery frame that holds the fabric and backing. Special effects can be created when 3D printing and embroidery are combined (e.g., [33, 34]). This was facilitated by a custom embroidery frame that interfaced with the researcher’s 3D printer [33].

A pattern, which is essentially a path, is then loaded and executed. Machine sewing involves a minimum of two threads. One thread is pushed through the fabric from the top to the back. The second is pulled up from below to catch the top thread and form embroidered stitches. These threads can be made of a variety of materials, and much of the computational textiles research uses conductive thread, though some have experimented with water-soluble thread [17], wire [63], fiber optics [24], or other specialty threads [19, 38, 90] or are conductive while also environmentally friendly [19], are becoming available.

Sewing needles traditionally are used to bring the thread down and through the fabric to create stitches. However, an alternative is to use a *cutwork needle* to cut the fabric. A traditional machine may have a wide variety of feet that facilitate movement of the fabric as well as different stitch types. Embroidery machines typically have less variety here, however one that has been used in some research is a *couching foot*, which is used to lay a thick strand of thread down on the fabric surface by zigzagging over it with a lighter weight thread. Couching has been used in many different domains, ranging from art [10, 39, 95] to sensing [75], for creating interactive conductive textiles.

Automated and machine embroidery techniques [58] can support the creation of shaped structures (e.g., [3, 11, 31, 33, 45, 63, 73, 83, 96]) and -interactive objects [30, 33, 87, 93], in addition to replicating long list of electronic capabilities (e.g., [1, 15, 32, 38, 45, 85, 89, 95]). Some work explores specific issues such as combining hard and soft components (e.g., [9, 61]). Given the importance of stitch orientation and density [88], embroidery research has also explored topics such as intelligent stitch path generation (e.g., [56, 86]). To our knowledge, this body of work has not considered the potential applications of these capabilities for tactile graphics.

However, research has explored other applications of sewing and embroidery in the accessibility domain [82], including a smart tablecloth for survey feedback during a performance [92]; a communication board for use during horseback therapy [72]; an assistive garment for audio localization [71]; a jacket that folds around a wearer who would otherwise have difficulty manipulating clothing to don it [55]; a tactile musical device for supporting sensory integration and stimulation [16]; and the FlexAbility project [12], which provides custom interactive wearables to people with disabilities. Textile and embroidered interfaces have also been used to support eyes-free interaction [29, 92]. For example, Giles *et al.* (2018) conducted participatory design with BVI crafters and artists to explore the construction of capacitive button-based tactile interfaces. Participants were given control over the creative process and made various functional and narrative objects. Artist Clarke Reynolds, who is blind, uses embroidery among other media in his work [80]. However, embroidered tactile graphics remain under-explored.

## 3 Manual Design of Tactile Graphics for Machine Embroidery

To understand the capabilities and needed considerations for embroidery to be a medium for conveying tactile information, the team conducted a mix of manual and machine design in two domains: children’s book illustrations and scientific diagrams. These examples were selected to capture a range of tactile needs– from conveying written information to spatial and context-rich details– and to surface the types of detail embroidery must meaningfully encode. Our goal was not only to assess embroidery’s material capabilities but also to examine how tactile graphics could be crafted to support legibility and haptic richness. Each example presented distinct design considerations and helped us build an understanding of how to shape embroidery for non-visual rather than visual experiences. We used the method described in [78] to produce Braille dots with the *Candlewick knot* texture provided by our embroidery software, *Embrilliance*^™^.

### 3.1 Children’s Book

Our first exploration was intended to help us understand the level of tactile richness needed to convey rich, visual information. To do so, we tested embroidery alongside additional techniques– experimenting with pre-loaded stitches and incorporating mixed materials where embroidery alone was insufficient to convey visual information. Our intent in exploring this wider space of possibilities was to ensure that our design recommendations would be unencumbered by existing technical limitations. Given that embroidered graphics have a long lifetime, it is appropriate to consider options that might require more manual labor or craft work, and develop guidelines relevant to both software designers and tactile designers– surfacing opportunities to move beyond repurposing of visually-oriented stitch libraries and begin developing stitch types and design systems explicitly tuned for tactile expressiveness.

The team analyzed a popular children’s book, *Where the Wild Things Are*, a book with detailed visual storytelling. Due to the length of the book, an abridged version was created. Following the “decode, access, communicate, and understand information” model of literacy [28], the team analyzed the book and identified ways of conveying information in scenes. Rather than simply trying to recreate the written story line, visual elements that conveyed information (e.g., a monster’s angry facial expression, a rough sea), were identified. These elements were coded into different categories: solitary (a key focus character or object), background (terrain, foliage, water), and pop-up (decorative elements designed to communicate depth), this is shown in Figure 3a. Properties of the elements that were conveyed visually (e.g. fuzzy fur) were noted, and the team explored different ways of doing so through tactile modes, prototyping with a variety of easily available pre-programmed stitches.

The explorations resulted in embroidered elements with different types and densities of fill. Yet it also surfaced difficulty with communicating visual information through visually-distinct stitches available on the embroidery machine. For example, our team did not find that the texture of the main character of the book was sufficiently distinct from that of scenic elements such as trees (Figure 3b and Figure 3c). When embroidery alone proved insufficient for communicating specific textures—such as fuzzy fur or cold stone—we augmented the designs with fabric overlays and 3D elements laser-cut from materials like felt, satin, and wood. These additional tactile elements follow recommendations from Norman of having the tactile representation be reminiscent of the real world object it conveys [65], and served as an exploratory probe into what unconstrained texture differentiation could be like in a tactile design. The addition of 3D elements draws from literature that suggests incorporating 3D elements enhances the tactile experience beyond what 2D content– even if texturally diverse– allows [6, 51]. The resulting prototype was a tactile book with embroidered, Braille text and embroidered, mixed-material imagery. Embroidery and other materials—such as satin for waves (Figure 2a), wood for a ship (Figure 2a), and felt for a fox’s fur (Figure 2b)–were used.

### 3.2 Scientific Diagrams

Our second exploration was intended to increase our understanding of the value of embroidered tactile graphics for scientific diagrams. Following standards for tactile graphic creation [59], we created two graphics. The first was a bar chart. We assigned textures to it using the optimization described in [78]. We noted that the lack of support for lines reduces the legibility of the axis and tick marks. The second, a heart, was assigned textures manually. A fairly complex graphic, the heart featured five textures along with lines.

We found that the glued-on Braille labels were difficult to attach to the edge of the cloth containing the Braille, creating a raised line region around each label that was sometimes jarring. In addition, the glue was not always reliable. We also found that, while the linear continuum used for texture assignment as presented in [78] performed well on graphics composed of a small number of distinct textures, more complicated graphics did not scale as neatly due to variability in the distinguishability of textures. This motivated our efforts to formalize factors influencing texture distinguishability described in Section 4.3.

### 3.3 Requirements for Designing Embroidered Tactile Graphics

Based on our explorations and prior work [78], we developed the following requirements for designing embroidered tactile graphics:

**Smooth backing** As described in [77], muslin cloth was rough enough to be a distraction from the embroidered textures. We found the satin backing used in the book to be far more pleasant to the touch.

**Lines are essential** For range of expressiveness, in both the book and scientific diagrams, lines are essential. Lack of lines in diagrams made regions harder to distinguish, and lines allowed the creation of complex textures such as the waves in Figure 2a.

**Stitch variations are the easiest way to express information** Despite the rich additions multi-material elements used in our book provided, the process of integrating them was highly-labor intensive and difficult to scale. Each visual element–whether fuzzy, smooth, or rough–required deliberate material selection and manual integration, such as fabric layering and hand-stitching.

**Range of expression is important for tactile legibility** While stitches are easiest to use, commercially-available software and machines prioritize visual aesthetics and differentiation of stitching. In this exploration, manually identifying distinguishable patterns required significant trial-and-error. Many visually appealing options resulted in designs that were imperceptible or insufficiently expressive. Careful stitch selection and verification, or better data-driven stitch selection, are essential.

This exploration surfaced a critical need to better understand how different types of embroidery feel, not just how they look, for embroidery to convey information as richly as mixed-material designs. Guidance is needed on what constitutes a suitable, legible texture for a specific purpose. We explore this gap in our next section (Section 4.1); categorizing properties of embroidered textures, and understanding what makes them distinctive and legible.

## 4 Categorizing Embroidered Textures

Our manual explorations of tactile graphics creation surfaced a gap in understanding of what a suitable, legible, and detail-rich texture could be when designing embroidered tactile graphics–with visually-distinct stitch patterns not corresponding to adequate tactile differentiation. This gap is reinforced by past work; Prescher et al. [70] note that tactile contrast cannot be reliably predicted visually; even if two textures look distinct, they may feel similar. To improve our understanding of the differences between embroidered textures, we embroidered samples of a variety of stitched textures and systematically classified them based on six complementary metrics capturing tactile differences. We classify the embroidered textures into three categories - textures for filled 2D areas, textures for lines, and textures for points.

### 4.1 Embroidered Texture Data Set

We compiled a set of fifty-five textures from the textures available in the Embrilliance embroidery software [25]. We removed a few that we observed to be highly similar, leaving thirty-four textures for filled areas and twenty-one textures for lines. Four of the area textures were also usable for points. Detailed definitions of stitches can be found in Embrilliance^™^, but we summarize some important examples here:

1. *Running stitch, Back stitch, and Stem stitch* are all ways of creating a line (Figure 5a). They can also be used to fill regions, such as when using a contour fill (Figure 5b).
2. *Satin stitch* creates a smooth fill for an arbitrarily shaped region using long or short parallel stitches. We included 10 different kinds of filled satin textures among our samples (Figure 5c). Satin stitch can also be used to create a wide line.
3. *Repeated motifs*, such as the cross stitch shown in Figure 5d, can be used to decorate a line or fill a region. We included a variety of cross stitch and other motif styles among our samples.
4. *Knots* can be used to create a line (Figure 5e), to indicate a point when a single knot is used, or for a filled region when these knots are used adjacent to each other to create a filled shape. An example is the Candlewick knot, which, when reversed, was the stitch we used to print Braille dots.

### 4.2 Sample Fabrication and Evaluation

Using the Embrilliance embroidery software [25], we uploaded an SVG (Scalable Vector Graphic) of a 25mm diameter circle. To evaluate area textures, we filled the circle with the texture. To evaluate line textures, we stitched the circumference of the circle with the texture. To maximize accuracy, we embroidered each texture onto a cream-white satin fabric with a cut-away stabilizer for backing, using black embroidery thread. These colors were chosen to maximize visual contrast because we used computer vision for the *whitespace* metric. We used a Janome MC 15000 with standard stitching tension to ensure consistent tightness for all stitch styles. Once the embroidery was done, we used a laser cutter to cut out a 52mm x 52mm square containing each region texture and a 30mm x 30mm square containing each line texture.

### 4.3 Quantities Measured

Textures are tactilely differentiated by a variety of features. For example, the contrast between two textures that are both smooth but differ in their height may be less than the contrast between a texture that is smooth and short and a texture that is rough and tall. As a result, we chose to use a mixture of numeric and nominal values to capture the differences between textures, all of which are not specific to any one embroidery machine or software unless otherwise stated. These include:

#### Number of Stitches (Nominal, Discrete).

The number of stitches used in a uniform region of a texture acts as an estimate of the density of the texture. A high number of stitches in a texture indicates a high density. This information was provided automatically by Embrilliance^™^, based on the calculated stitch path for each texture (Figure 6a).

#### Weight (Nominal, Continuous).

Weight measures the amount of thread in a pattern. It differs slightly from number since the length of thread used in a stitch will vary. Thus, a stitch that uses more thread will have a greater weight at the same number of stitches as a stitch that uses less thread. For each texture sample, we measured its weight using a gram scale with a precision of 0.001*g*, as shown in Figure 6b. The type of yarn and fabric were consistent across all samples and should not effect the distribution of weights.

#### Height (Nominal, Continuous).

The height of a texture describes how much the thread in each stitch pushes out of the fabric creating a tactile sensation of depth. This can help distinguish similar textures that share a border (e.g., Figure 6c). To measure the height of each of the textures, we used calipers with a precision of 0.1*mm*.

#### Whitespace (Nominal, Continuous).

The amount of whitespace in a pattern is a rough estimate of the degree to which the underlying satin is felt when touching the pattern. To calculate the white space, we took photos of each stitched pattern under the same camera angle, distance, and light conditions. We converted the photo to black and white (Figure 6d), cropped it to the stitched circle, and calculated the ratio between the patterns’ pixels and the total pixels.

#### Direction (Categorical).

The direction indicates the primary flow of the stitches in the texture and only applies to textures over areas. Direction is an important component in designing tactile graphics because it helps users understand the orientation of an area. We determine direction through orientation, flow, and texture. For example, in Figure 7, the texture on the left would be *diagonal* and *horizontal*. The texture in the middle would be *grid*. The texture on the right would be *unpatterned*. For each sample, a sighted researcher analyzed the sample and assigned it a direction of one of five categories: *Unpatterned* textures with a randomized or inconsistent flow (9%), *horizontal* textures where stitches are aligned in rows (24%), *vertical* textures where stitches are aligned in columns (21%), *grid* textures that are aligned with both rows and columns (15%), and two categories of *diagonal* textures that flow either from the *top left to bottom right* (15%) or from the*top right to bottom left* (42%).

#### Motif Category (Categorical).

Motifs describe a classification of embroidery stitches common across embroidery craft practices. We assigned each swatch a motif category based notify type defined in the Embrilliance software[25]. We anticipate very similar stitch patterns would be producible in other softwares, but may be named differently. Areas consisted of eight motif fill categories: *candlewick*, *knot*, *larger motif*, *cross-stitch*, *satin*, *decorative*, *squared*, *blank*. Line textures consisted of eight motif categories: *cross-stitch*, *satin-fill*, *satin-border*, *french knot*, *free standing lace*, *contour echo*, *stipple*, *run*.

#### Stitch-Complexity (Categorical).

Across motif categories, we additionally classify the complexity of a stitch pattern based on the shape of the repeated stitches in each texture. For each sample, a sighted researcher assigned a stitch-complexity category based on the following criteria: *Simple* (55%) if the texture is composed of only straight lines (e.g., cross stitch, horizontal lines); *Repetitive* (39%) if there is a repeated, spaced-apart motif; *Complex* (6%) for all other patterns.

#### Custom Texture Rankings (Nominal, Categorical).

BVI team members contributed to ranking the set of textures. The textures were ranked in order of perceived roughness, with higher values meaning rougher textures. We repeated this custom ranking twice on two sets of textures. The first set included all fifty-five textures. In the second set of textures, we removed 8 very similar textures: three very rough and five very smooth textures. This results in two sets of measurements.

### 4.4 Summary

Through our process of categorizing textures, we identified seven tactile properties that influence how embroidery textures are differentiated and perceived. *Number of stitches*, *weight*, *height*, *whitespace*, *direction*, *motif category*, *stitch complexity*, and *perceived texture* were all identified as properties that influence the tactile distinctiveness of embroidery stitches. These differentiators form the basis of our optimization approach for selecting distinctive textures, a key component of our pipeline for producing tactile graphics, covered in the next section.

## 5 Selecting Textures for Non-Visual Differentiation in Embroidered Tactile Graphics

In the prior section (Section 4), we identified key attributes that influence perceptibility and distinctness of embroidery stitches. In this section, we build on these learnings to automate the design of texturally-differentiable tactile graphics. Specifically, we focus on scaling the ability to produce graphics with clearly-denoted regions, each representing different types of visual information. To make such graphics legible, neighboring regions must be significantly distinct so that the texture has a clear change at their borders. Additionally, each region of the graphic should have a unique texture so the reader can use the sensation of the texture to recall the region’s meaning. Notably, regions do not need to be continuous. Multiple sub-regions may share a texture to denote a consistent concept. For example, in a diagram of a living cell, multiple regions may represent one type of organelle using the same texture. To achieve this automation of texture selection, we create an optimization algorithm for selecting and assigning textures to tactile graphics with diverse regions.

### 5.1 Optimizable Model for Non-Visual Differentiation in Tactile Embroidery

We model embroidered tactile graphics as a set of items *i* ∈ ℝ that form a graph, where each vertex is a region, line, or point in the tactile graphic. Any area not covered by a region, line, or point will not be embroidered (i.e., white or empty space). Each item *i* neighbors a set of other items that it touches. We refer to the neighbors of an item *i* as ℕ*_i_*. Each region is assigned an embroidery texture *t_i_*. Items can be either areas covering a 2D portion of the graphic, lines following a path along the graphic, or points. The type of item restricts the set of appropriate textures since some textures can cover areas, follow specific paths, and clearly denote a specific location on the graphic. To optimize a tactile graphic, we must assign textures to each item such that there is sufficient contrast between neighboring regions and across the whole graphic so that the reader can identify each distinct item and its boundaries. This representation is similar to the model of tactile maps presented by Hofmann *et al.* [42].

### 5.2 Optimization Method

The goal of the optimization is to assign a texture *t_i_* to each item, *i*, of the graphic. We use a stochastic hill-climbing algorithm for our optimization, a configuration that has been used in other tools for textile design [43]. Starting from a random assignment of textures to item, we iteratively modify the texture-item mapping. We evaluate each graphic with an objective function that aims to maximize neighboring and overall contrast between textures. We store each new mapping in a population of discovered graphics. In each iteration, we select a graphic from this population with a bias for the highest-performing graphic. We then select a method to modify the mapping of textures to item, which is expected to improve the objective score the most given a set of heuristics (Figure 9). This method was implemented with the OPTIMISM framework [40] described by Hofmann *et al.* [41].

We use fifty-four textures (thirty-four region and twenty line textures). The “Run-double” line texture was not included in the optimization - after printing some sample graphics using that texture, we chose to omit that texture because it was too thin and flat to be identified as a line on a graphic. The five textures used for points were selected from a subset of the region textures, with the same calculated values. When a color is used for a mix of element types, we assign a texture from a smaller set of textures that are valid for all relevant element types.

### 5.3 Objective Function

The optimization process is driven by an objective function that assesses the quality of a specific mapping of regions to textures for a given tactile graphic. The objective function is the weighted sum of a set of objectives that evaluate individual criteria. Maximizing the score of the objective function will increase the legibility of the embroidered tactile graphic. Using the OPTIMISM framework [40, 41], each objective is a function that returns a value between zero (poor-performing graphic) and one (high-performing graphic). The distribution of each objective score is defined by a Gaussian-bell curve that tends towards one when the objective returns a value near a given target value *α*. The parameters of each objectives curve are listed in Appendix D.

The following describes the four categories of objectives that make up this objective function:

#### Neighbor Contrast.

For a graphic to be legible, it is critical that all neighboring items contrast and can be distinguished by touch. Contrast can be achieved by differences across multiple texture qualities (see Section 4.3). For instance, a dense and heavy texture can be clearly distinguished from a less dense and lighter texture. Similarly, varying motifs and stitch complexity can distinguish two textures. There is no single correct way to distinguish textures for any two neighboring regions.

We evaluate the contrast between two textures as the proportion of different texture qualities to the full set of seven texture qualities. Two categorical qualities (i.e., direction, motif, stitch-complexity) differ if the textures do not belong to the same category. Two nominal texture qualities (i.e., number of stitches, weight, height, proportion of whitespace) differ if the absolute value of their difference is greater than the range values for that quality in our data set divided by the number of items in the tactile graphic being optimized.

To ensure that no two neighboring items of the graphic use indistinguishable textures, we define our first objective: *minimum contrast between neighboring textures*. This objective will linearly approach one as the minimum contrast between neighboring textures approaches a target alpha value that we default to five based on empirical tests.

To increase the contrast across all neighboring items in the graphic, we define our second objective: *maximize average contrast between neighboring textures*. This objective will linearly approach one as the average contrast between neighboring textures approaches the target value of six, which we determined empirically through pilot tests.

#### Overall Contrast.

Similar to neighboring contrast, a quality tactile graphic will have a diversity of high-contrast textures. Consider a reader examining two portions of the graphic with two separate fingers; even if the two items are not neighboring, the textures should be distinct so the reader can recall which finger is touching which item.

We use the same method for evaluating the contrast between two textures as we used to assess neighbor contrast. To evaluate the overall contrast of a graphic, we include two additional objectives that measure the minimum contrast across the graphic and the average contrast across the graphic. Based on empirical tests, we default the target minimum contrast as four and the target average as five. These target values are lower than the default values of the neighbor contrast objectives because it is more important that immediately adjacent textures have contrasting textures so the items can be distinguished.

#### Stitch-Motif Diversity.

While textures can contrast based on many metrics, past work showed that assigning textures with a wide distribution of stitch types produces more comprehensible graphics [78]. This may be because, while contrasting stitch metrics such as weight and height create tactile differences, each unique motif is more identifiable and can be readily recalled by a reader.

To ensure a wide distribution of stitch motifs, we include an objective that is the proportion of motifs used in a item-texture mapping over the number of motifs available (i.e., sixteen motifs in Embrilliance [25]). Additionally, we want to ensure that items are equally distributed across each motif. For example, the satin motif of textures contains ten textures that are all very smooth, and the candlewick and knot motif of textures contain six textures that are all very rough. While there is a variety of contrasting textures in this category, a tactile graphic primarily composed of satin textures and only one candlewick texture, which tends to be rough, would be difficult to interpret. To ensure a balance between motif categories, we add two final objectives: one for the count of satin textures, and one for the count of candlewick and knot motifs. The target of these objectives are one because, ideally, there will be only one texture of each motif in the graphic.

### 5.4 Summary

Together, these objectives allow for the creation of an optimization algorithm that assigns stitch-type regions in tactile graphics based on measurable differences in tactile properties. By considering both neighboring and overall contrast objectives, the algorithm aims to improve the overall textural diversity and distinctiveness of the graphics when read by touch. The model supports different element types– areas, lines, and points. This approach creates an automated, scalable way to do texture assignments across tactile graphics.

## 6 Pipeline for Creating Embroidered Graphics

**Figure 1:**
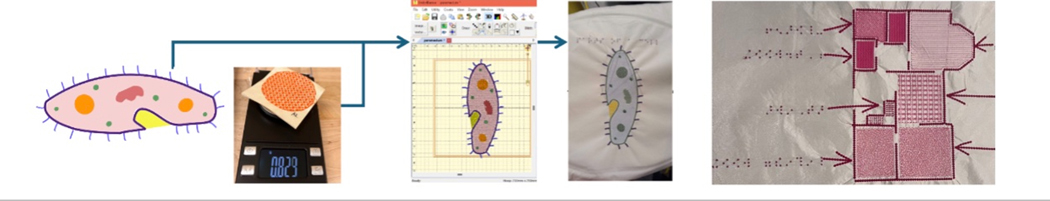
Pipeline for embroidered tactile graphics. Given a prepared diagram such as a picture of a cell, and data about textures (such as weight and direction), our optimization algorithm assigns textures to the diagram. The textures are manually applied to the SVG using commercial embroidery software and then embroidered. Next, Braille is added, completing the graphic. On the right is a second example, a floorplan map, stitched using the same pipeline.

**Figure 2:**
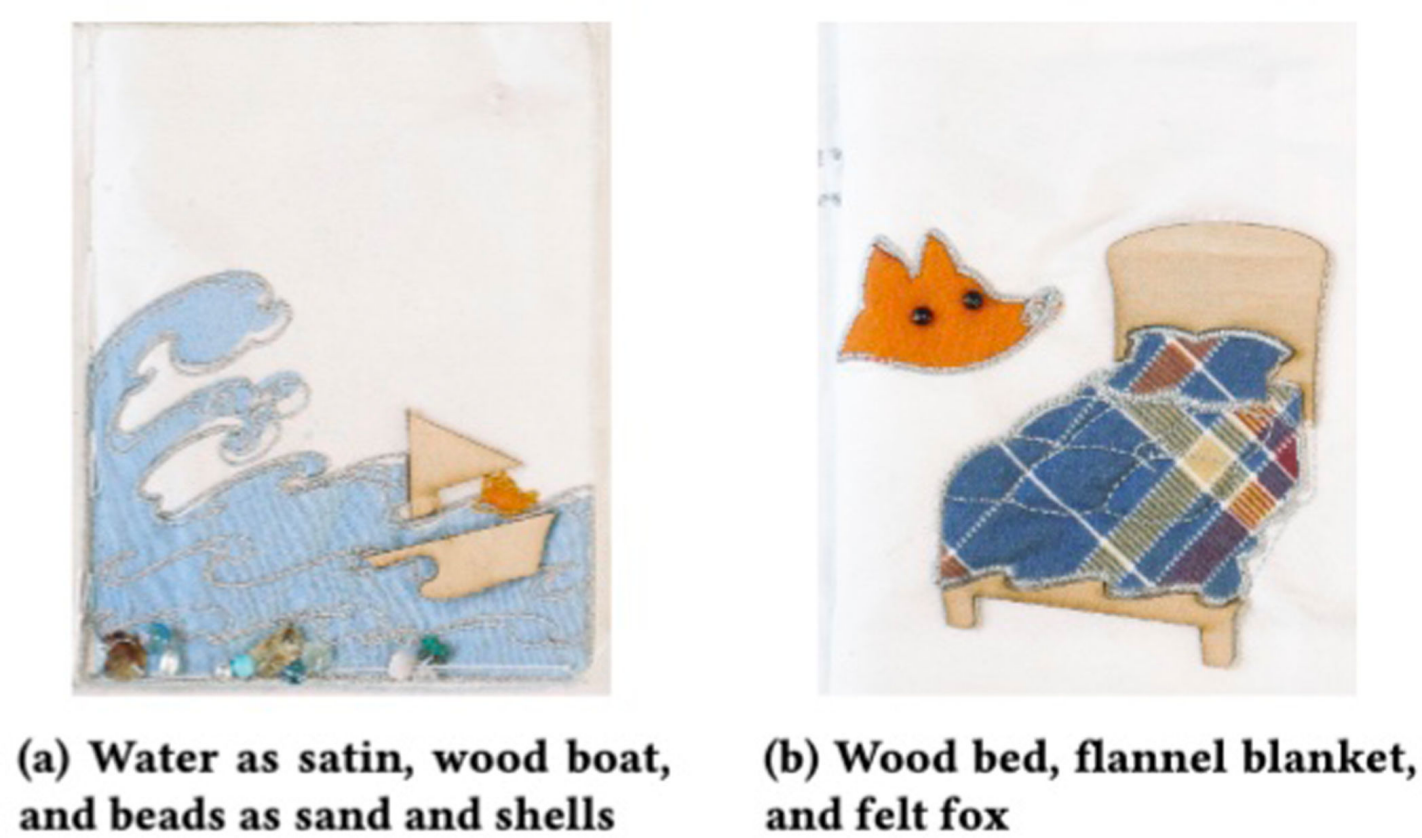
Tactile book page with multi-material visual details

**Figure 3:**
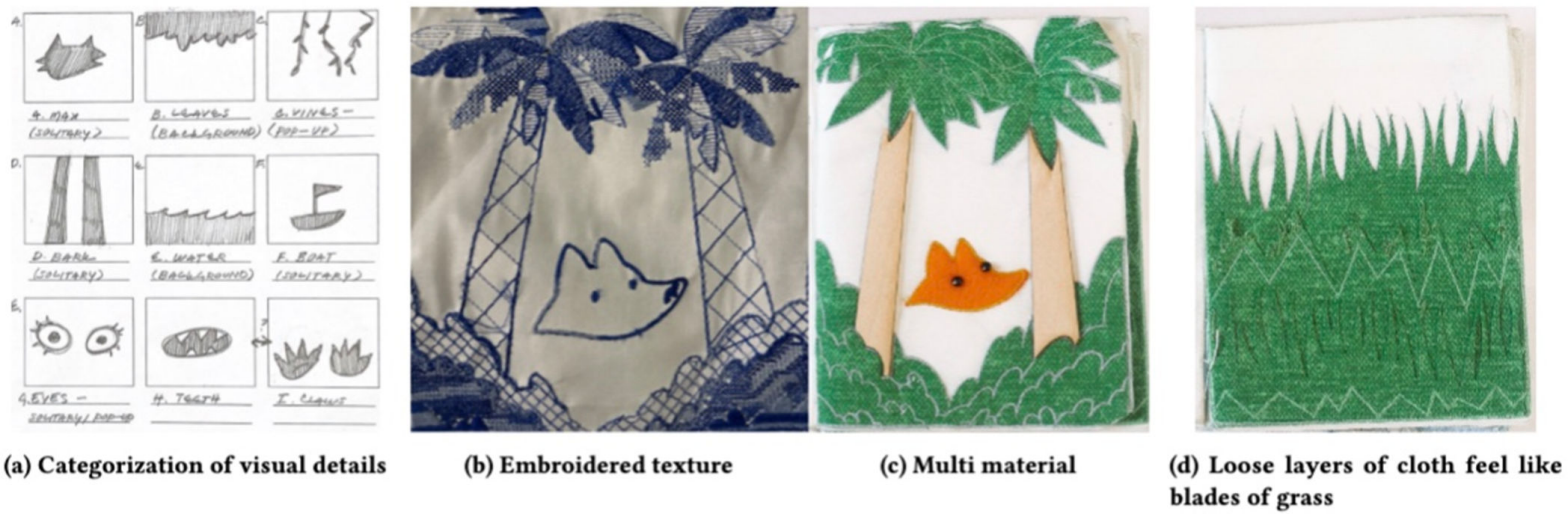
Design process, iterations in identifying and testing representation of visual detail. Final two images shows use of multimaterial and multidimensional visual details

**Figure 5:**
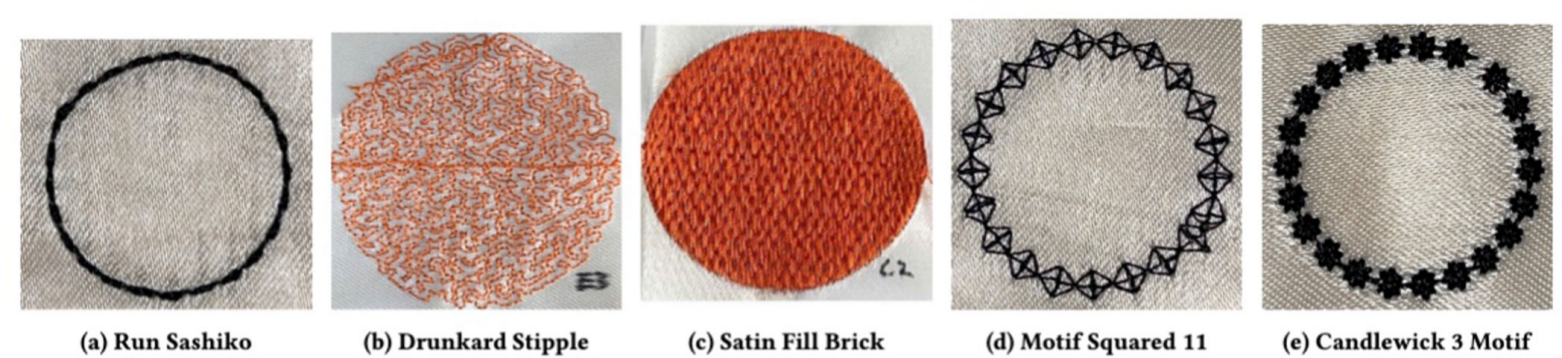
Example textures and their uses for lines and fills. Texture names are based on Embrilliance^™^for reproducibility.

**Figure 6:**
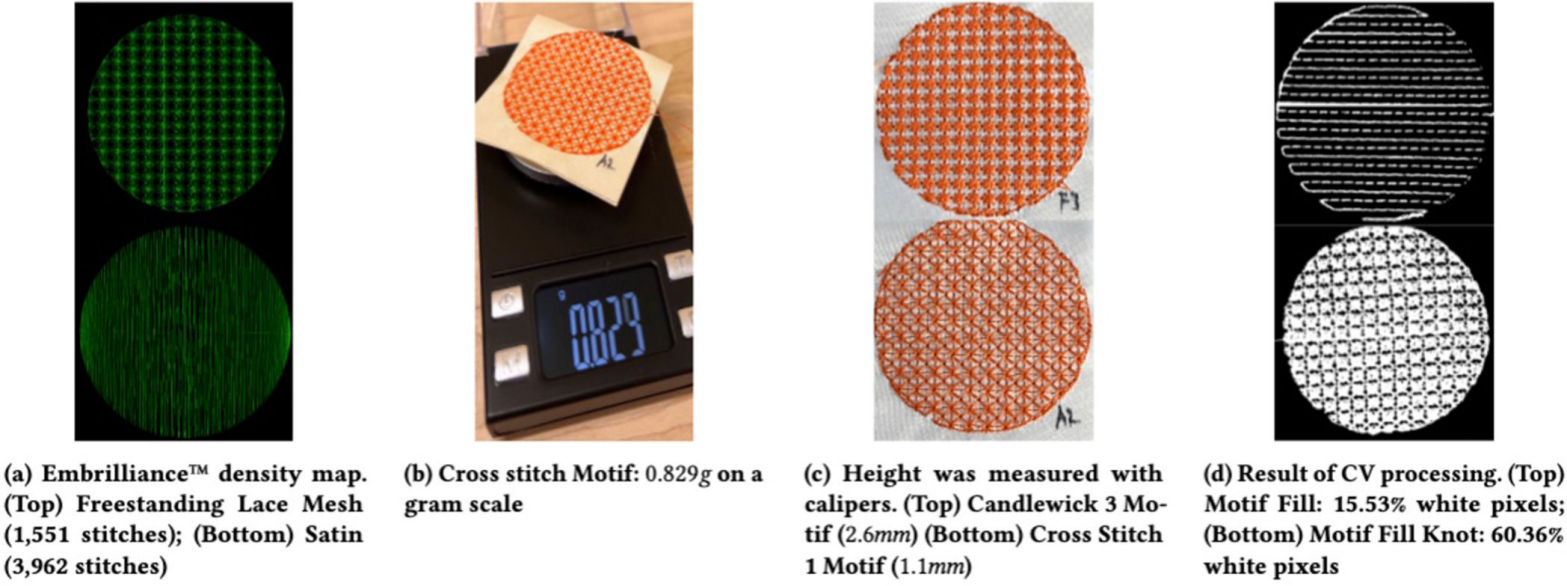
Measurement strategies. Results are summarized in Table 1.

**Figure 7:**
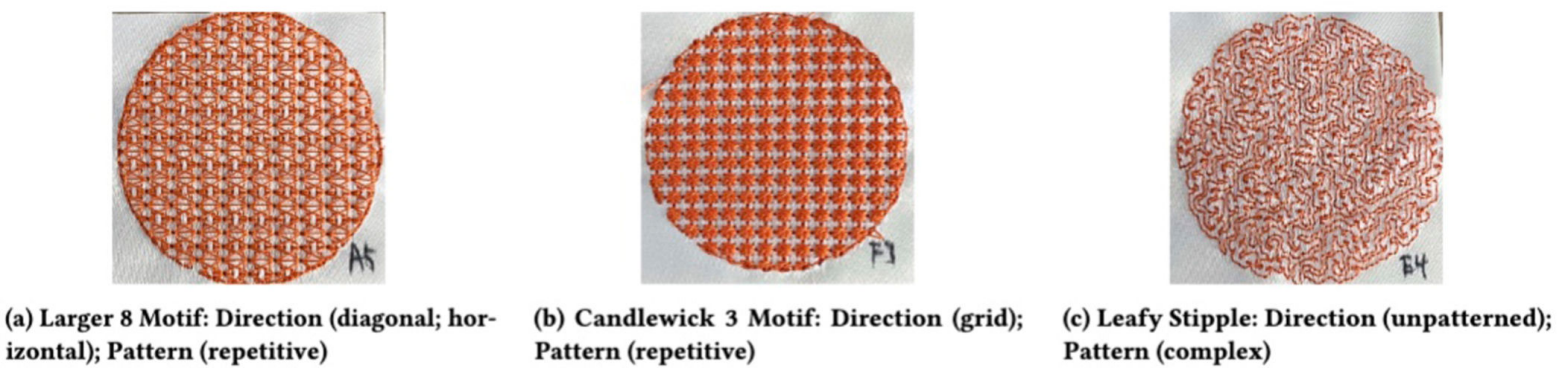
Examples for pattern and direction

**Figure 9:**
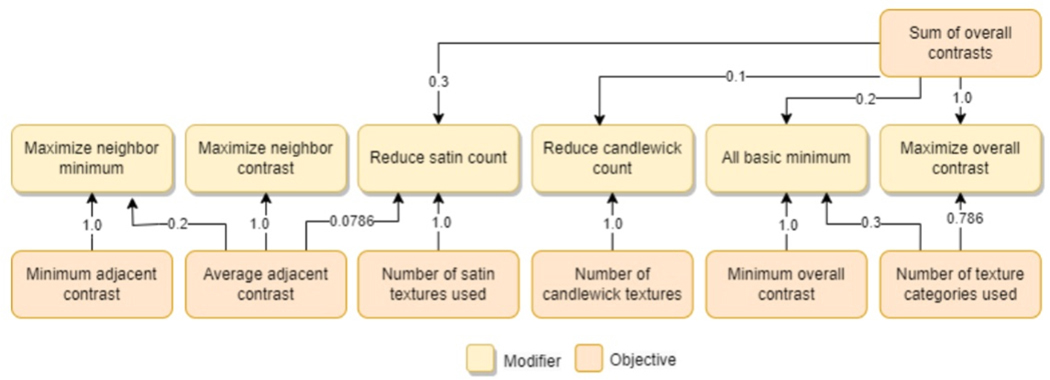
Heuristic map representation of the relationships between ways of modifying a mapping of textures to regions (i.e., modifiers) and objectives that measure different qualities of the mapping based on the OPTIMISM framework [40, 41].

We build on our optimized texture assignment algorithm to develop an end-to-end pipeline to map visual graphics into physical, embroidered tactile graphics. We start with an SVG file that includes the different parts of the tactile graphic. This SVG file is fed into an optimization algorithm that assigns stitch textures to each region of the graphic. The SVG file is then loaded into stitch editing software (Embrilliance^™^), where the textures chosen by the optimization algorithm are manually applied. Then, the graphic is printed using an embroidery machine. This involves securing it in the embroidery frame, printing the stitch file, then inverting it in the frame, and printing Braille labels. Post-processing involves washing, ironing, and cutting any hanging threads. More details about each of these steps are described in the following subsections. Figure 1 summarizes each of these steps.

### 6.1 Requirements for the SVG File

Our pipeline assumes that an SVG file containing the tactile graphic to be embroidered has already been created manually. SVG is an XML-based vector-based image format. It may include *paths* (which can be used to represent lines as well as filled areas); *basic shapes* (such as closed polygons, circles, and rectangles); and *text* (which we do not support). Paths that are the result of path operations (e.g., intersection, difference) are supported by our pipeline, but if using Embrilliance^™^, some path operations might be removed after loading the SVG file and will need to be reapplied in the software. Advanced techniques such as clipping, masking, and compositing are also not supported by our implementation. These elements can have a *color*; a *fill* (in the case of regions); a *stroke color* (in the case of lines); and an ID (a string label for that element). For paths with a fill color and a different stroke color with a non-zero stroke width, our pipeline detects both the filled region as an area and the surrounding border as a line. We add the following requirements for an SVG to work with our pipeline:

1. Any related regions should have the same color values for fill and stroke. For example, a ribosome (one of the parts of a cell) may appear multiple times, so all ribosomes in the graphic must be given the same color. The optimization will assign all regions of the same color to the same texture.
2. Because a point is just a tiny circle, it should be distinguished from a filled region by including the word “point” in the ID for that region.
3. If a border is created using a path object without a fill, that region must have the phrase “border” in the id for that region to be recognized as a line.

**Figure 10:**
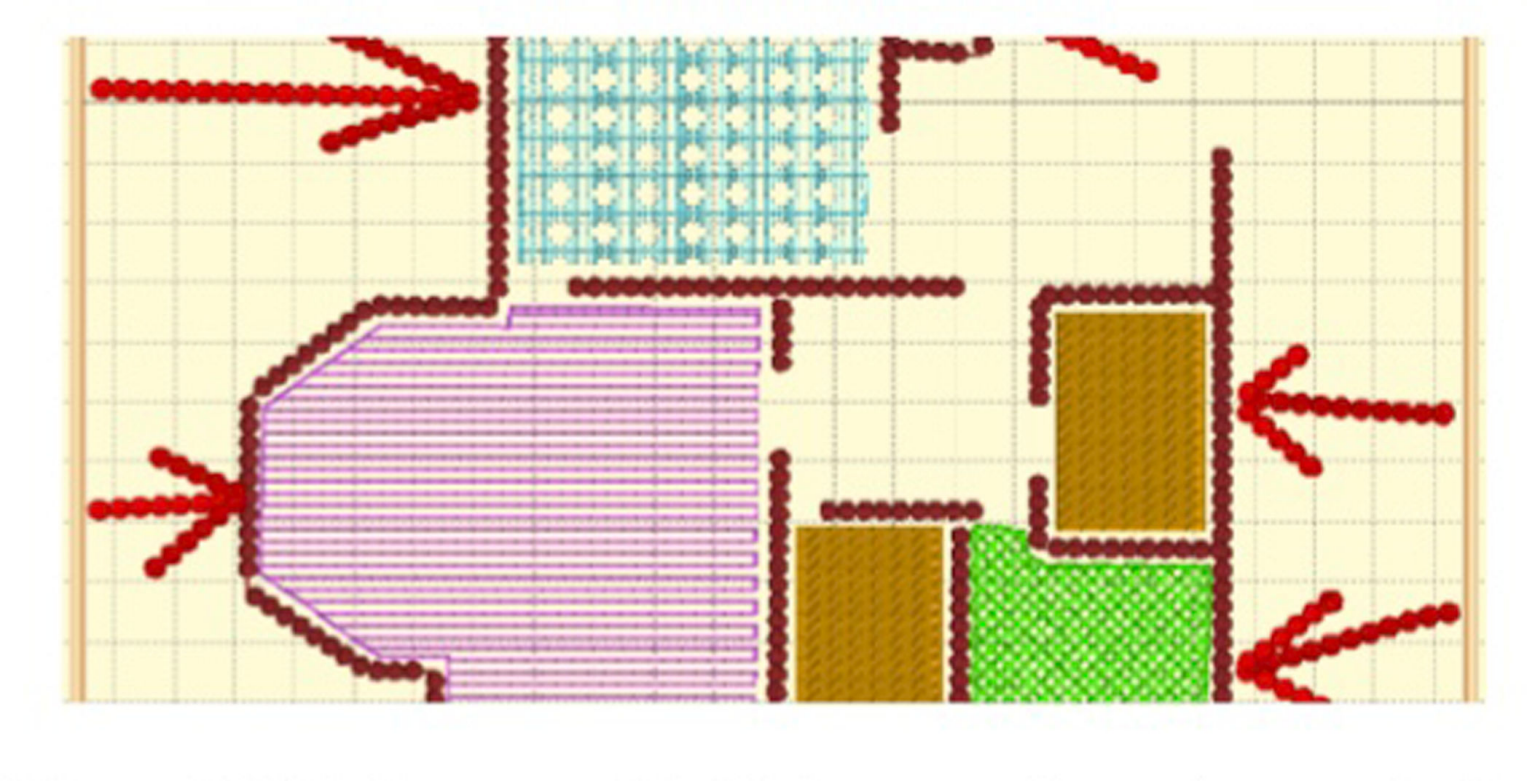
Whitespace added between walls and rooms in the embroidered floor plan print file

Through our iterations on embroidered designs, we also learned that adding border lines between regions, with a small amount of white space, could help make neighboring regions more distinguishable (Figure 10).

We use an XML parser to extract each element, its associated color, and whether that element is a line, point, or filled region. In addition to this basic information, our optimization algorithm needs to know which elements are adjacent to each other.

To estimate adjacency, we use a bounding box of each element *e*. For lines, the bounding box is computed assuming the line has a thickness of one pixel. Two elements are considered adjacent if their bounding boxes overlap or share a border. This information is stored as a set of regions and the optimization algorithm described in Section 5.

There are some limitations of this approach, such as if the shape is shaped like a U or is rotated. Many shapes may not be exactly rectangular, so the bounding box for that shape would include some parts that are not part of the shape. This could result in some shapes that are not adjacent being classified as adjacent, but will not hinder the quality of the graphic as it will only hold those two shapes to a higher standard of contrast.

### 6.2 Printing the Embroidered Graphic

Once the optimization algorithm has assigned textures to each region in the graphic, a stitch file is created in Embrilliance^™^by loading the SVG file and assigning the textures to each region. This stitch file is then loaded into a machine embroidery machine (we used the Janome S9 and the Janome Horizon) via a USB drive, where the file can be printed onto a fabric secured in a hoop. We used satin fabric as the background because of its smoothness. To reduce puckering in the graphic during printing, we used a stabilizer on both sides of the graphic. Water-soluble sticky stabilizer was used on the top side of the satin so that it could be washed off after printing to retain its smoothness and a non-water-soluble stabilizer was used on the reverse side of the fabric to add stability to the overall graphic.

#### 6.2.1 Creating Legible Braille.

Braille is composed of individual units called “cells”, which are created using different combinations of six dot configurations (see example of full six dot cell). We aimed for the embroidered Braille to follow the Braille Authority Guidelines. We determined empirically that the size of the dots in the SVG file needs to be smaller than the guidelines while the spacing between them needs to be larger than the guidelines because the actual embroidered dots end up being larger than in the SVG files. Table 2 shows how the Braille Authority guidelines compare to the sizes in the SVG file and the measurements (collected using a digital caliper) in the final embroidered graphics and embossed graphics. We were unable to reduce the width of the individual dots more than what we already did without the stitches getting printed inconsistently and loosely. We assigned Braille dots to the bobbin side of the motif fill of candlewick knot 3, with Embrilliance^™^settings of 3.4 mm width and 3.5 mm height at a density of 2.7 mm between each line of stitching (determined empirically based on feedback from an author experienced with Braille use).

Since we used the bobbin side of this stitch for the Braille, the Braille needs to be printed on the reverse side of the side the main graphic is printed on. To do this we first created a program that translates any alphanumeric phrase into an SVG containing a mirror of standard visual Braille. Additionally, stabilizers are used on both sides of the fabric to prevent stitches from sinking, which would make the Braille illegible and increase errors where the thread catches on the embroidery machine.

#### 6.2.2 Printing the Graphic.

Our printing process is a multi-step process in which the main part of the graphic is first printed, the fabric is then flipped, and then the Braille is printed on the reverse side of the fabric. In total, print times varied from about 2 to 4 hours. Post-processing of the tactile graphic took up to 45 minutes. These times vary on the complexity and size of the graphic.

**Figure 12:**
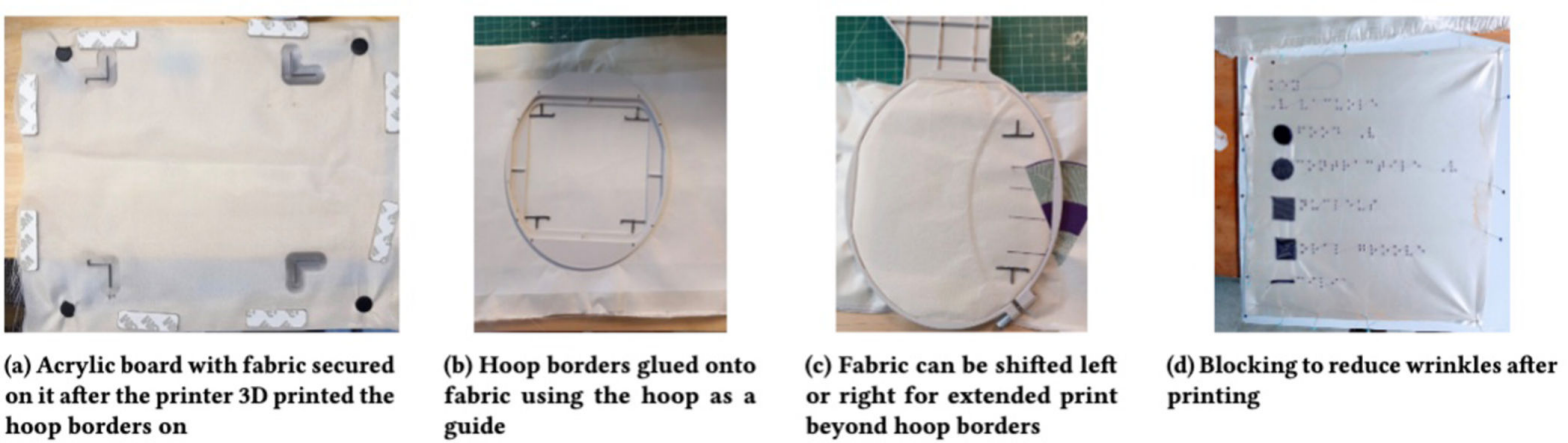
Images of important components of the printing process

To make sure the Braille is correctly aligned onto the graphic after the image is flipped, plastic borders are printed onto the fabric before any embroidery is done. The borders demarcate the edges of the hoop. We experimented with multiple approaches. Initially, we printed the borders directly onto the fabric using an acrylic jig and magnets to secure the fabric properly (Figure 12a). However given the manual nature of the overall process, we ultimately found that stretching the fabric in the embroidery frame and then manually gluing 3D printed borders onto it was just as effective and more flexible (Figure 12b). For example, manually gluing borders allowed us to switch machines and hoop sizes without having to create a new jig. The first method was slightly more accurate; however, for label placement, this difference was not noticeable. Future work could explore developing a double-sided embroidery frame to achieve maximum accuracy (since, in that case, the cloth would not need to be removed and re-stretched).

Some of our graphics were larger/wider than the available hoop size when labels and associated arrows were added. In this case, we printed the graphic in multiple batches. For example, first, the main part of the graphic is printed, and then the fabric is adjusted in the hoop to print the Braille on the left/right side of the graphic. To align the fabric correctly when adjusting it to the left or the right, we designed a second type of hoop border marker that is “T” shaped (rather than “L” shaped) that allows for printing more of the tactile graphic to the left or the right. The bottom two borders are “T” shaped and the upper two borders are an upside down “T”. Figure 12 shows how these borders work. For example, in Figure 12c, after printing the main part of the layers graphic, the fabric removed from the hoop, shifted to the right, flipped, and placed back in the hoop, using the two leftmost “T” shaped borders to align the fabric in the hoop - the Braille labels to the left of the layers graphic could then be printed.

#### 6.2.3 Post-processing of Embroidered Tactile Graphic.

After the entire graphic is printed onto the fabric, the fabric is washed in cool water to remove the water-soluble sticky stabilizer. It is important that all of the sticky stabilizer is removed to prevent any sticky residue that will make it difficult to read the graphic. Even though we used stabilizers on both sides of the graphic, there was still puckering in most of the graphics. We tried two different methods to reduce this puckering after the printing was complete. (1) Blocking involves pinning the wet fabric onto a corkscrew board or a piece of cardboard and then waiting for the fabric to dry. When pinning the graphic, the pins are placed all around the graphic in a way that stretches the graphic and reduces wrinkles (Figure 12d). (2) Another method we tried is placing the wet fabric between two cloths and then ironing over the piece of cloth. By placing a cloth under the graphic as well, the stitches are better preserved without being damaged by the iron. Both these methods helped with reducing the puckering but did not fully remove it.

## 7 Legibility and Comprehension Evaluation of Embroidered Tactile Graphics

To understand how well embroidered tactile graphics can be understood, and whether there are certain types of tactile graphics that embroidery is well suited for, we conducted a small, six person study using four different tactile graphics. Because we wanted to test understanding using both quantitative and qualitative methods, we focused on complex maps and diagrams about which we could ask questions relating to comprehension. We also asked participants to think aloud during the study and conducted a qualitative analysis of their experience.

### 7.1 Selection of Tactile Graphics

Comprehension of tactile graphics is often dependent on the style of tactile graphics presented and the information domain it presents. For example, a reader will need to gather different information from a map of their college campus than from a diagram of a bacteria from their biology textbook. To evaluate whether our embroidery pipeline produces results that are legible across a variety of domains, we produced tactile graphics of two diagram types–navigational maps and scientific diagrams–each with two domains, resulting in four categories of tactile graphics. The study included: diagrams of an organism; layers of a geological object; a geographical region; and an indoor floor plan.

**Figure 13:**
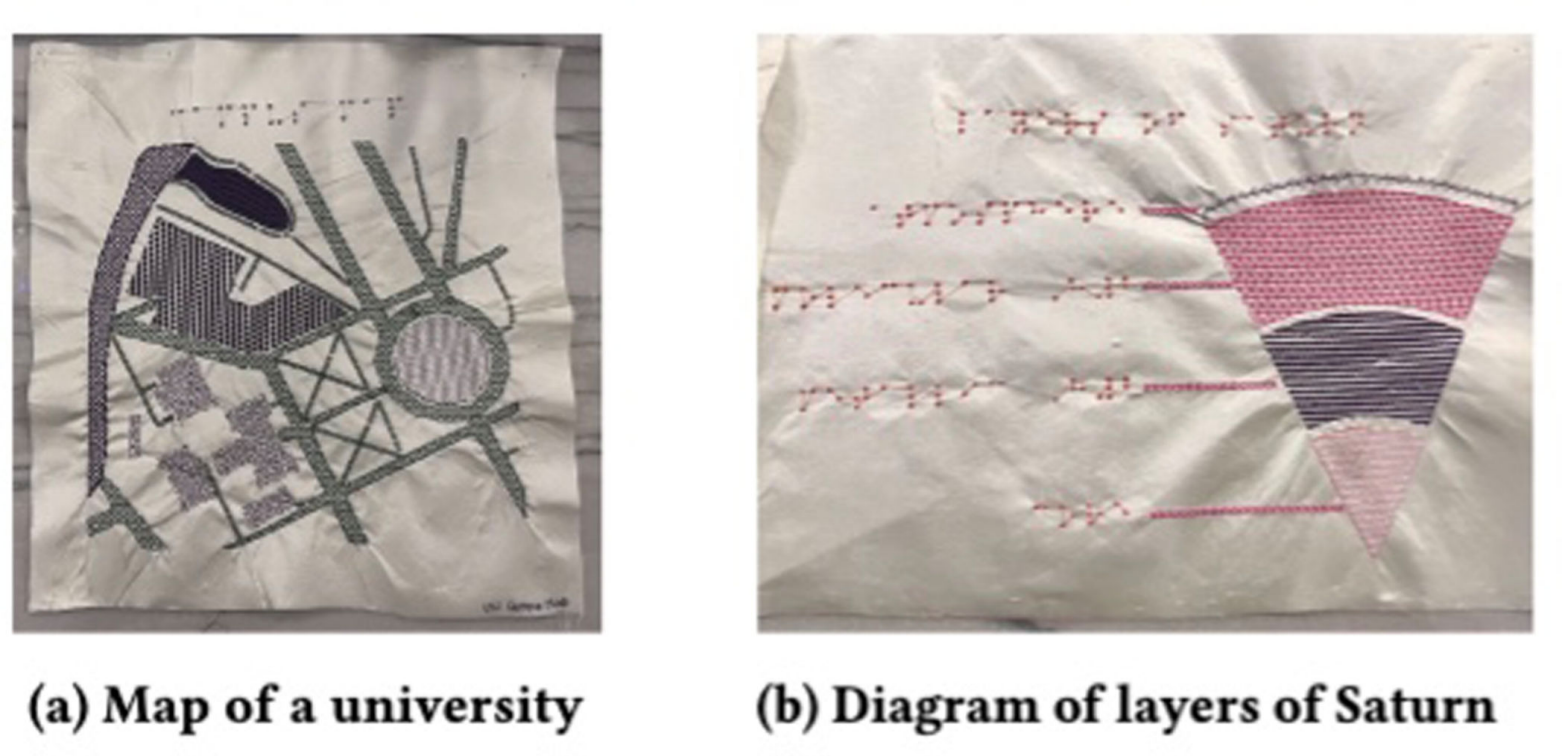
Sample embroidered graphic used in our study

For each category, we designed an SVG file. For example, Figure 13a shows a university campus map and Figure 13b shows layers of a planet. For consistency, we designed all of the SVG files for the tactile graphics ourselves, referencing best practices for tactile graphic design [59]. The complete set of graphics used in our study can be found in Appendix B.

### 7.2 Methods

**Figure 14:**
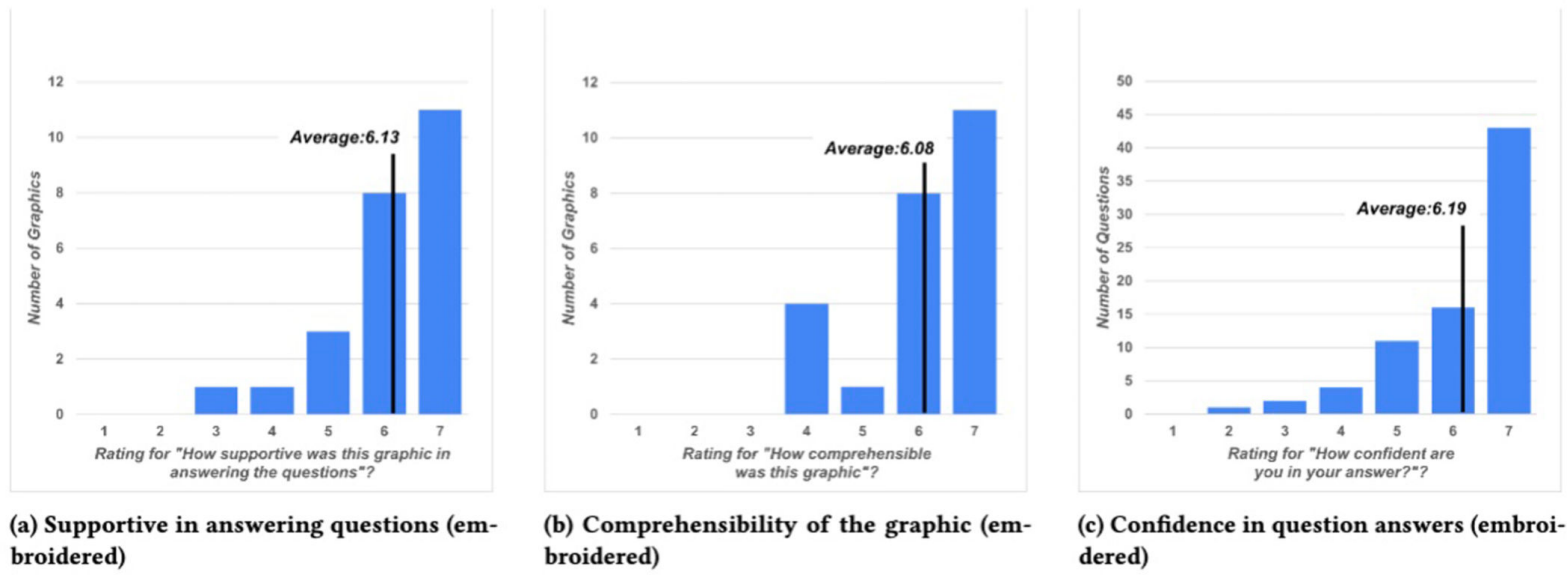
Participant Ratings

We conducted a think-aloud study to assess the legibility of the tactile graphics. Participants explored each tactile graphic while verbalizing their process with prompts from the researcher. Participants were not limited in this exploration time and were not given an expected time for each graphic by the researchers. When the participants determined they had thoroughly reviewed the graphic, the researcher asked them a standardized series of questions that depended on the graphic. Participants were allowed to revisit the graphics while answering these questions. After answering the question, the participant rated their confidence in the answer using a seven-point Likert scale. After all the questions for the graphic were asked and answered, the researcher asked the participant to rate the graphic’s readability, comprehensibility and confidence (See results in Figure 14). They also asked about the support offered in answering questions by the graphic, and aspects of the graphics that helped or detracted from understanding.

The comprehension questions for each graphic were designed to elicit a variety of ways of interpreting a tactile graphic such as comparing the size of different regions, examining the spatial relationships between regions, searching for specific labels, and identifying regions by specific criteria and labels. The questions required no prior domain knowledge and could be answered correctly using only information in the graphic. Each question was designed to have one unique correct answer, ensuring there was no ambiguity in our assessment of the participants’ comprehension. The comprehension questions are presented in Appendix A.3.

Participants provided verbal informed consent to participate in this research. Each session took less than 90 minutes, and participants were compensated $40 per hour for their time and any additional transportation costs. One researcher conducted each session in person, while another joined virtually on Zoom to record the study and take notes. Both researchers had prior training to ensure that the research methods were accessible to participants the researchers [57]. Across all participants, we included the following accommodations. The presenting research avoided the use of visual pronouns and prepositions (e.g., “over there”), described directions verbally (e.g., “directly in front of you”), and verbalized their actions (e.g., “I’m passing you a tactile graphic...”). Automatic captions were enabled to assist the researcher taking notes over Zoom. These methods were approved by the last author’s institutional IRB.

The data collected during this study included a video recording and a transcript of the study. We also recorded the responses of participants for each of the questions and any observations as they explored the graphics using a Google Form. After the studies, we compiled the numerical responses (e.g., confidence and comprehensibility ratings), computed summary statistics for each measurement, and created histograms showing the results. We also compiled a set of stand-out quotes, grouped by feedback about the Braille, textures used, and overall experiences in each graphic, that we could use to evaluate the quality of embroidered tactile graphics.

### 7.3 Participants

Six BVI participants, each with varying experiences with tactile graphics, participated in a 90-minute user study. All participants were US-based adults and were experienced with reading Braille. Participants were recruited through posts made in online communities. Our recruiting materials specified that we were looking for “blind or low-vision individuals who have experience with reading tactile graphics and Braille.” We screened for Braille literacy because our diagrams were labeled in Braille, rather than a proxy for tactile diagram understanding. Table 3 contains demographic information about each participant, and self reported experience levels with Braille and tactile graphics.

Participant A used embossed tactile graphics frequently in high school but has not used them as frequently in college because the university does not provide them in a timely manner. Of all the participants, participant B had the most experience with tactile graphics, using them frequently in high school and college, and has used tactile graphics for diagrams of different systems in physics and for other kinds of systems. He uses tactile graphics every week, if not daily. Participant C has not used many tactile graphics other than some tactile graphics for architectural drawings and web design layouts. Participant D has mostly interacted with artistic designs that are on shirts and some embossed graphics. Participant E stated that they “come from a different country where tactile graphics are not as available” but has “participated in different studies and I started to introduce myself to different forms of tactile graphics in the recent years.” They mentioned that the graphics they had interacted with are about trends, graphs, and social science topics. Participant F mentioned that she used tactile graphics in high school for a variety of concepts in math courses. She stated that she interacts with tactile graphics “as often as I get my hands on them”, for example at a tactile art show.

### 7.4 Results

#### Participants Understood Embroidered Graphics.

Participants were able to answer 70 questions out of a total of 78 questions correctly (89.74%). Figure 14 shows participant rankings of comprehensibility and supportiveness of the graphics, as well as their confidence in answering questions about the graphics. As can be seen, confidence was somewhat spread out. This could reflect differences in the complexity of the images.

#### Simplicity Helps With Readability.

Based on the feedback of the participants, it is better to use fewer textures when possible and more empty space if the region does not need to be filled in, aligning with existing recommendations [59]. For example, for the floor plan embroidered graphic, Participant A mentioned, “*each area has different feelings, but then I think it will be helpful to have just the border lines instead of both the borders and the filled regions*” (Participant A) . At the same time, some participants found that the diversity of textures used to differentiate regions in embroidered graphics helps explore without having to constantly refer to Braille labels. It also offers the opportunity to encode more information into the graphic such as density. For example, Participant C stated that “*there is a difference in the textures of various elements that are here which is helpful...*” (Participant C) . Even though our results indicate that most participants matched textures correctly during their explorations of the graphics, participants also mentioned feeling unsure about differences between textures at times, especially for graphics complex enough to need legends. Participant B mentioned that the “*textures are not super distinct—they are different, but [it] takes a moment to calibrate*” (Participant B) .

#### Braille was Difficult but Possible to Read.

Because the texture of the Braille is different from standard Braille on embossed tactile graphics, and because the Braille dots were more spaced out than traditional Braille, all participants had some trouble with reading the Braille. Embroidered Braille, being produced from thread rather than embossed paper, was much softer than traditionally produced Braille. For example, one participant mentioned that, “*this [embroidered] Braille is very difficult to read because the dots are large and spaced out.*” (Participant F) . They reported that these slight spacing differences make reading slower than traditional Braille. However, as the study went on, all participants except participant D reported feeling more confident and being able to more quickly read the Braille. Participant D stated that “*I can’t really tell you what these say, but I could probably guess if I knew the names*” (Participant D) . Participant A mentioned that they “*...struggled on matching keys in the embroidered [graphic,] but if I practice on it I should be able to find it easier*” (Participant A) and that “*now I’m used to feeling the Braille on this graphic...now I’m getting used to it*” (Participant A) . Participant C stated, “*Braille quality is nice but I need more practice*” (Participant C) . More practice could be helpful, but it may also be possible to improve legibility in the future.

Overall, despite some of the limitations of embroidered tactile graphics, participants expressed excitement toward the embroidered tactile graphics and stated the approach has the potential to compete with embossed paper tactile graphics. In addition to feeling that embroidered tactile graphics are less “monotonous” than typical approaches (i.e. embossed graphics), participants also expressed that the potential for tactile depth and diversity allows embroidery to afford more options to creatively represent elements in a tactile graphic. Participant B mentioned that embroidered tactile graphics may be especially useful in more complex diagrams, where embossed tactile graphics can get confusing: “*There’s a lot of instances, though, where you need a more complex diagram and embossing no longer does the best job of that...you could actually simplify that by using embroidered textures*” (Participant B) . For example, for the embroidered tactile map, they said, “*the intersecting roads and paths and stuff like that...can be made very clear by a [embroidered] diagram like this, where you just simplify each all of those like intersecting lines and things like that*” (Participant B) . Another mentioned that embroidered graphics might have potential in domains such as mechanical engineering, natural sciences, and geology because “*you have more opportunity to play with different textures to represent different parts*” (Participant E) . Participant F felt that embroidered graphics would work better in more creative or artistic settings - “*if it’s something that feels like it has kind of an artistic feel to it, embroidered is better*” (Participant F) .

#### Results summary.

Our evaluation showed that, for the six participants in our study, embroidered tactile graphics across distinct domains (maps, scientific diagrams) were comprehensible and legible. Participants also raised design consideration for legibility such as the need for simplicity and importance of spacing. Importantly, participants described how some of the texture attributes we identified (e.g. density) could encode information. Participants were excited about the potential of embroidered tactile graphics, expressing benefits such as expressivity and broad range of textures in contrast to existing methods such as embossed graphics.

## 8 Discussion

Our exploration of embroidered, tactile graphics surface a set of design considerations ensuring these graphics are created in a legible, detail-rich way. These considerations emerge from lessons learned through our manual explorations of embroidery as a tactile graphic medium, categorizations of texture, end-to-end pipeline of creating distinct tactile graphics, and participant evaluation.

### Textural richness of embroidery should be measured and defined, not assumed.

Our manual exploration of the tactile books showed that visually-different textures were not sufficient at communicating detail-rich visual information Section 3). This was confirmed through our categorical analyses of available embroidery textures, which found more effective ways for measuring texture differences than by appearance. The participant evaluation emphasized the importance of some of these attributes, such as white space and density, in conveying information and bolstering comprehension. Our work contributes a structured approach to measuring and assigning textures across perceptual dimensions (e.g. density, height, directionality). Existing design tools should surface these attributes alongside visual details of the stitching to better communicate the tactile expressiveness of various stitches.

### Overall embroidery design is just as important as the details.

In our manual exploration of scientific diagrams, we found that holistic details such as the material that the diagrams had just as much of an impact on legibility as stitching (Section 3.2). The concept of overall legibility was explored further in our design of the texture-assigning automation tool, which found that both distinctions of local, neighboring textures and overall textural diversity was important. This was confirmed in our evaluation, where participants negatively described the impact of too many similar textures on the overall comprehension of the diagram. Designers should think about textural details not only at an element-level, but in the context of a full graphic– including other represented elements and even materials it is printed on.

### Embroidered tactile graphics should strive to be engaging, not just legible.

The participants of our evaluation described how embroidered tactile diagrams allowed for less monotony and more creative affordances than other alternatives such as embossed graphics. While our evaluations centered on comprehensibility and legibility due to the fact-oriented nature of our test materials (map, science diagrams), embroidery is already being used as a medium for data physicalization of personal materials [91]. As a participant described, embroidery could be useful in creative and artistic settings. In mediums where emotional tone and scenic detail are critical–such as tactile books– texture could play a key role in unlocking the nuances of this in an expressive way. If we can better understand and assign textures based on intended meaning, rather than solely distinction, embroidery could become a promising tool for creating tactile graphics that are not just informative, but deeply expressive.

## 9 Conclusion and Future Work

We have presented samples of how embroidery can be used to create tactile graphics, surfaced important considerations for the design of legible and haptically rich graphics, and presented a pipeline for creating machine-embroidered tactile graphics. We evaluated outputs of our graphics across two different domains, and participants who used them were able to answer most questions correctly and with confidence. Based on feedback from participants, we believe that embroidered tactile graphics have the potential to represent more complex graphics than embossed graphics because of a potentially larger array of different textures available through different stitch types compared to that of embossed graphics.

### Comparison of embroidery to traditional methods.

Future work should compare embroidered tactile graphics to traditional methods of tactile graphics to assess whether there are differences in legibility. Although the durability of embroidered graphics and their overall comprehensibility make a strong case for their use in some circumstances, a comparative study would help to evaluate and document trade-offs of their use. Based on our preliminary study, we expect that there are scenarios where embroidery is preferred as a medium, such as in more artistic contexts, but further work is needed to evaluate this across different domains and types of graphics.

### Explore legibility of Braille.

Creating embroiderable Braille that was legible and universally-comprehensive was difficult. We found that factors such as texture and spacing influenced legibility. In future work, the spacing in the Braille could be further reduced with custom stitch path generation code to improve readability. Our settings were based on the limitations of the machine we used, but it is possible that the spacing between dots and cells could be further reduced with other machines, or by experimenting with changing dot print order or stitch paths.

### Improve accessibility and reduce manual steps.

With the pipeline described in this paper, the steps of creating the SVG file for the tactile graphic, and putting together the stitch file after the optimization algorithm are performed manually. To further streamline this process for creating embroidered tactile graphics and make the process more accessible to the general public, it would be ideal to automate creating the SVG file based on an image and creating the stitch file given the textures returned by the optimization algorithm.

Additionally, some of the tools and interfaces we used (e.g. embroidery machine screen) are not accessible to BVI audiences, limiting the ability of disabled makers to create their own embroidered, tactile graphics. Future work should examine making the process of designing embroidered tactile graphics accessible end-to-end to a broad range of users.

### Increase user control.

Even though all the embroidered tactile graphics were readable and participants answered most questions correctly, based on the results from the user study, there are some changes to the embroidered graphics that could be made to further enhance the readability. The optimization approach could be updated to allow users to specify preferences, such as whether to fill regions. The objective function could also be changed to include a term trying to minimize the overall number of textures used, or to maximize the amount of uncovered space, when a texture and border provide redundant information. This would help to address participants’ wish to have few textures to improve clarity.

### Explore new application spaces.

Finally, now that we have demonstrated that embroidered tactile graphics are viable to produce and legible, future work should explore the unique value of these graphics in settings where other tactile graphics may not be robust enough, or available enough, to merit their use. For example, recreating the visually-intricate children’s book we explored with mixed-materials in Section 3 using our automated texture selection tool, and subsequently deploying it in-field, would be an interesting next step. The durability of embroidered graphics and the wide array of tactile feelings offered by embroidered textures are a promising alternative to traditional tactile graphics.

## Supplementary Material

Video presentation

## Figures and Tables

**Figure 4:**
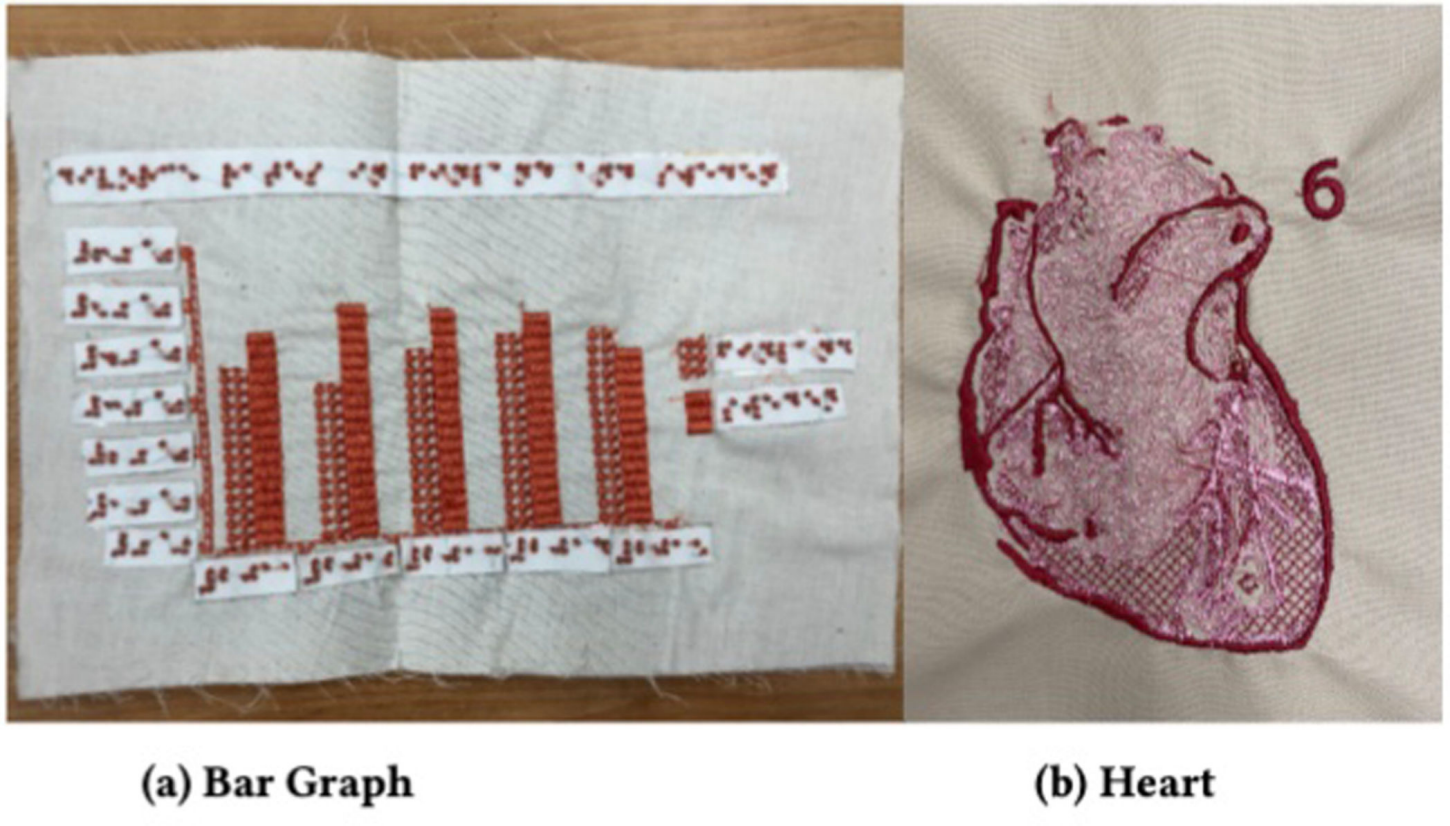
Experiments with scientific diagrams

**Figure 8:**
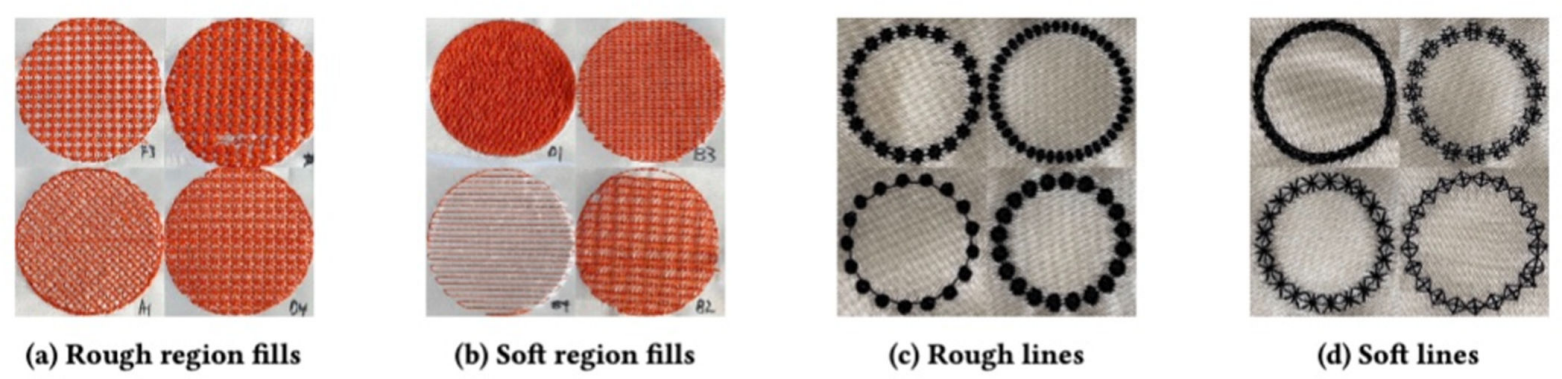
Textures rated as very rough and very soft. In order from top left to bottom right, textures are (a) Rough regions: Candlewick 3 Motif; Candlewick 1 Motif; Free Standing Lace; Sashiko Contour Echo; (b) Smooth regions: Satin Fill Diamond 3; Decorative 39 Motif; No Motif; Larger 27 Motif; (c) Rough lines: Candlewick 3; Candlewick 1; French Knot; Candlewick 2; (d) Smooth lines. Run Chain; Knot 5; Cross Stitch 1; Squared 11.

**Figure 11:**
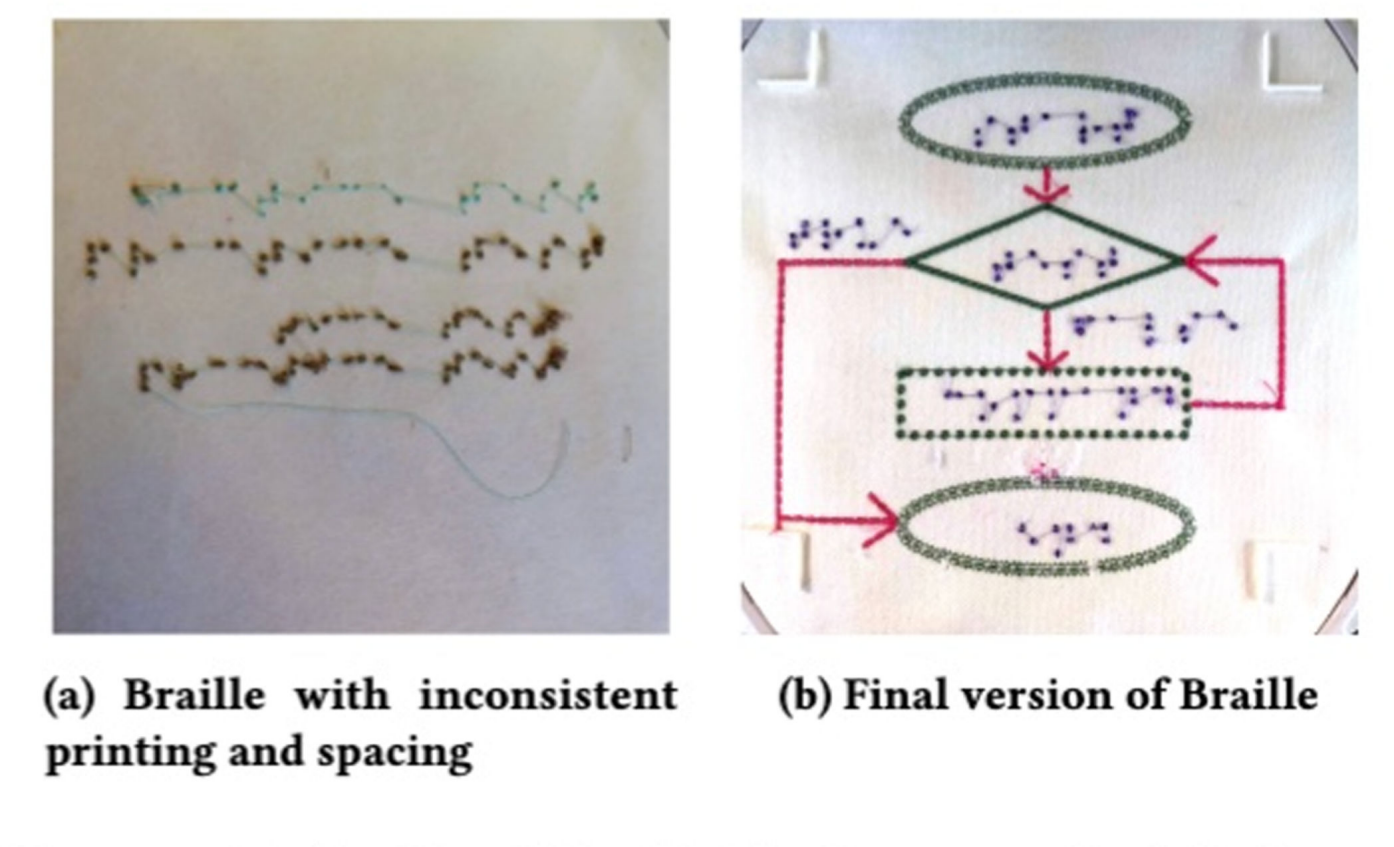
Embroidered Braille. The image on the left shows the thread bunching up and the dots being printed inconsistently when trying to reduce the spacing and size of each dot, and the right shows the final Braille.

**Figure 16:**
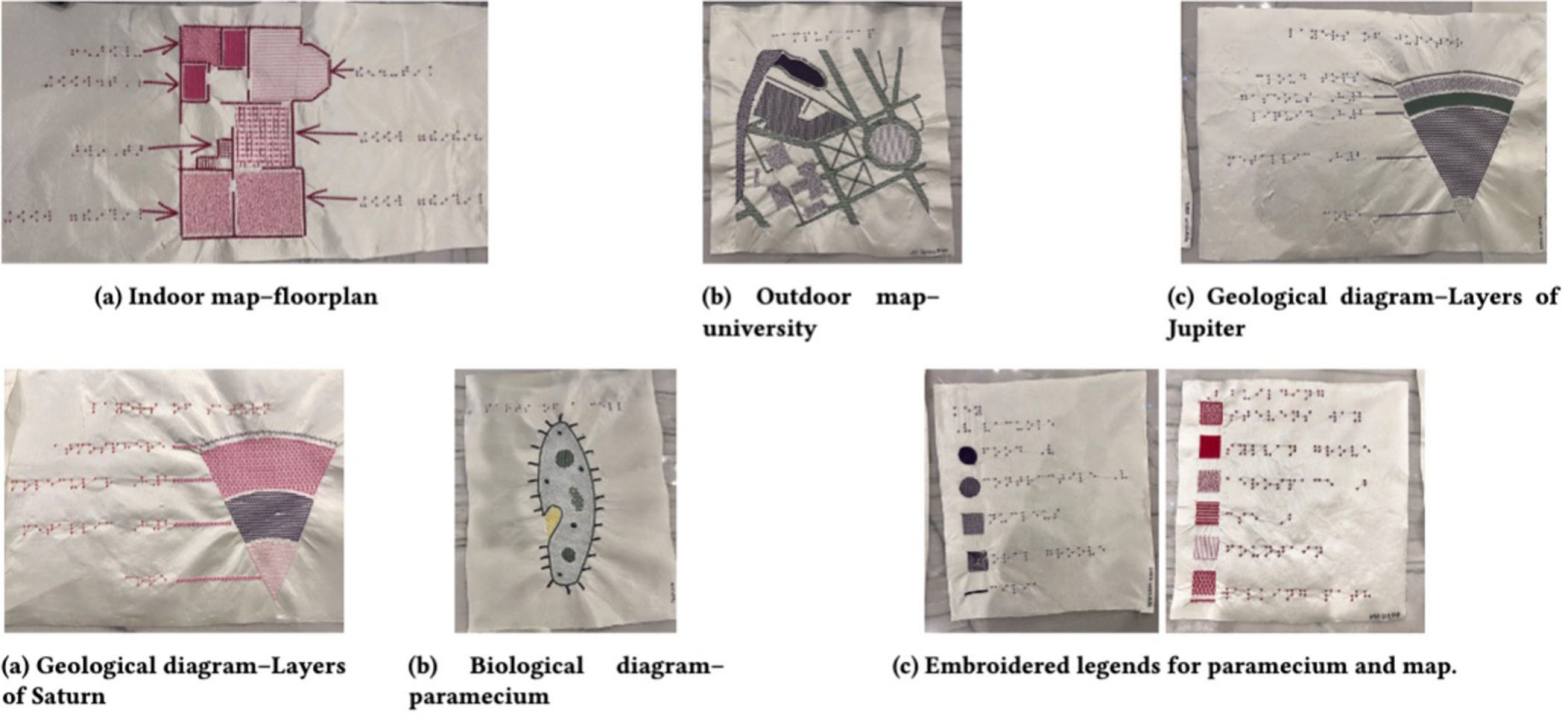
Embroidered graphics used in our study

**Table 1:** Measured values for regions and lines.

Measure	Region			Line		
	A	M	R	A	M	R
Stitches	3322	3336	1149–6299	371	322	56–1035
Weight	1.13g	1.06g	0.7– 1.82g	0.26g	0.25g	0.22–0.36g
Height	1.57mm	1.5mm	0.7–2.6mm	0.98mm	0.99mm	0.6–1.6mm
Whitespace	42%	44%	14–61%	24%	22%	6–25%

A=Average; M=Median; R=Range.

**Table 2:** Table showing measurements for the embroidered vs. embossed Braille.

	Diameter of each dot	Distance between dots in the same cell	Distance between corresponding dots in adjacent cells
Braille Guidelines	1.5–1.6mm	2.3–2.5mm	6.1–7.6mm
Created Braille SVG files	1.016mm	4.572mm	12.09mm
Printed embroidered Braille	2.21mm	2.28mm	9.13mm
Embossed Braille	1.61mm	1.87mm	5.40mm

**Table 3:** Demographic information for each participant about the nature of blindness, and self-reported experience levels with Braille and tactile graphics on a scale from 1 (least experience) to 5 (most experience).

Participant	Age of vision loss	Nature of vison loss	Reported Braille experience	Reported tactile graphics experience
A	Congenital	Unknown	3	3
B	Around 6 or 7	Unknown	5	5
C	Congenital	Total	3	3
D	Congenital	Central	3	3
E	Congenital	Total	3	3
F	Congenital	Total	5	5

**Table 4:** Standardized factual questions for the tactile graphics

Graphic	Type	Comprehension Questions	Correct Answer

Paramecium	Biological Diagram	Locate the oral groove.	See Figure 16
		Identify all the contractile vacuoles in this cell.	See Figure 16
		Find and identify what the smallest part is in the cell.	Food Vacuoles
		What is the name of the hairs sticking out of the border of this cell?	Cilia

Layers of the Planets	Geological Diagram	Name the layers from top to bottom in Saturn.	atmosphere, molecular h2, metallic h2, core
		Identify and locate which planet has the layer of molecular hydrogen.	Saturn
		Is the metallic hydrogen layer larger in Jupiter or Saturn?	Jupiter
		Which layer is the largest in Jupiter?	metallic hydrogen

University Campus Map	Outdoor Map	Locate the Sylvan Grove.	See Figure 16
		Where are the aerospace buildings in relation to the cse building?	Below

Floorplan	Indoor Map	What is below the living room?	dining room
		Which room takes up the least space?	closet and/or bathroom
		Find the kitchen.	See Figure 16

## CCS Concepts

- Human-centered computing → Accessibility.

## Footnotes

1 We use Unified English Braille system, Grade 1 for our work

## A User Study Questionnaires

### A.1 Demographics Survey

At the start of the study, each participant was asked the following questions:

- What kind of experience have you had with tactile graphics in the past?
- What kind of tactile graphics have you used?
- Is there a particular setting/domain in which you have more experience with tactile graphics?
- How often do you use tactile graphics?
- What are some things you look for in a quality tactile graphic?
- On a scale from 1 to 5 where 1 is very little and 5 is very much, how much experience would you say you have had with reading tactile graphics?
- On a scale from 1 to 5 where 1 is very little and 5 is very much, how much experience would you say you have had with reading Braille?

### A.2 Think-Aloud Graphic Exploration Prompts

As the participants explored each tactile graphic, they were asked the following prompting questions:

- What are you noticing about the graphic as you’re feeling it?
- What are you feeling?

### A.3 Tactile Graphic Comprehension Questions

See Table 4

### A.4 Tactile Graphic Review Questions

After each tactile graphic was complete, the following questions were asked:

- On a scale from 1 to 7, where 1 is not at all and 7 is very much, how confident are you in your answer? (Given for each comprehension question)
- What, if anything, did you find helpful about this graphic?
- What, if anything, was confusing about this graphic?
- On a scale of 1–7 where 1 is not at all and 7 is very much, how much did this graphic support you in answering the questions?
- On a scale from 1 to 7 where 1 is not at all and 7 is very much, how comprehensible was the graphic?

### A.5 Post-Study Review Questions

After all tactile graphics were complete, the following reflection questions were asked:

- What feedback do you have on the Braille labels in both types of tactile graphics?
- What are the most important aspects of a tactile graphic for you? How did these different approaches measure up to those priorities?
- What is your feedback on the textures used in these graphics?
- What, if anything, did you like or dislike about the embroidered tactile graphics?
- Do you have any other general comments/reactions/feedback you would like to share?

## B Photos of Each Tactile graphic

See Figure 16.

## C Tactile Graphics standards referenced

Each of the above graphics was constructed with attention to existing tactile graphics guidelines authored by the Braille Authority of North America [59]. Specifically, the moon graphic utilized cross sections to represent depth in 2D (guideline 2.11), the lines pointing to descriptors in the floor plan were designed with reference to standards for lead lines and arrows (guideline 3.4), and separately printed legends were used for clarifying meaning when the graphic itself was too large to support lead lines as was the case for the paramecium and campus map (guideline 5.7, 5.8).

## D Weights used in the Optimization

The following weights for each of the seven objectives were empirically determined and used in the optimization:

*Category diversity* : 7.0

*Neighbor contrast - Mean* : 11.0

*Neighbor contrast - Minimum* : 1.0

*Overall contrast - Sum* : 11.0

*Overall contrast - Minimum* : 5.0

*Overall smoothness*: 3.0

*Overall roughness*: 1.0
